# Supplementary material for: Gender imbalances among top-cited scientists across scientific disciplines over time through the analysis of nearly 5.8 million authors
Source: PLoS Biol. 2023 Nov 21;21(11):e3002385. doi: 10.1371/journal.pbio.3002385 (PMC10662734; doi:10.1371/journal.pbio.3002385)
Supplement: Attachment — Submitted filename: pointbypointplosbiology.docx [file pbio.3002385.s001.docx]

Editor comments

You'll see that all three reviewers are broadly positive about your study, but each raises a number of concerns that will need to be addressed before further consideration. Reviewer #1 asks if the study was preregistered, notes some short-cut citations, wants you to report some additional data, asks about the universality of some academic posts, and wants more detail on how authorship position was considered. S/he also makes some recommendations for improved dataviz and suggests some additional analyses. Reviewer #2 wants more rigorous formal stats to support your assertions of differences between fields and through time; s/he has some further requests for clarifications and extra analyses. Reviewer #3 is somewhat more guarded; s/he wants you to rectify a mismatch between the claims and the choice of academic subfields, recommends pooling small subfields to make numbers of researchers in each more comparable, questions the robustness of your gender classification tools (especially if it has subfield-specific problems), and challenges the relevance of your “country” analysis.

Reply: We have addressed all of these comments and concerns. Please see detailed responses below to each reviewer comment.

Academic Editor comments

"I agree with most of the reviewers comments, each brings up important points and none of them are unreasonable.

Reply: We fully agree and addressed all of them.

"I see where the authors are coming from, no, not everything is going to need to be hypothesis tested. However, as reviewer #2 points out, descriptive stats are important for making some of the points that authors would like to make. Post hoc tests are typically used for these sorts of "additional analysis" ensuring an overall lower significance rate. Of course the n's here are quite large so I would assume that no matter which analysis is done, it will be significant so I can certainly see why the authors chose this route.

Reply: We have added some statistical testing results so as to complement the numerical evidence presented.

"I also agree with the reviewers about the https://namsor.app/. Much like many of our analyses, the presence of a black box component (either a commercial app or an antibody kit) needs to be treated carefully. The authors simply need to address this issue, it is not disqualifying, but should be treated more carefully.

Reply: We have added additional language about the caveats and error rate of NamSor and also discuss the error rate found in the 100+100 random sample of authors that we selected for in depth assessment/verification.

"I would also ensure that all RRIDs were added to the manuscript. There are RRIDs for software tools and databases that are appropriate here and are not included."

Reply: We have tried to include complete information for software and databases. If anything is still missing, we are happy to add this as well.

Reviewers’ comments

Reviewer #1:

The article "Differential correction of gender imbalance for top-cited scientists across scientific subfields over time" is very well written and an important contribution in the field of gender imbalances in the scientific community. However, there is a notable omission in the presentation of results, which could enhance the overall impact of this study. There are a few concerns that need to be addressed before publishing in PLOS Biology.

Reply: Thank you for the kind appreciation of our work. We have addressed the suggestions raised, as detailed below.

Methods:

*	Has the study been preregistered? If so please provide the link and/or DOI.

Reply: The study was not officially pre-registered. In fact, we did write a detailed protocol and did follow it to collect and analyze the data, but we did not deposit it in public. We have added that “This is a descriptive, exploratory analysis of a science-wide large bibliometric dataset (not pre-registered).”.

*	Particularly the methods section contains quite some short cut citations. Linking to the previous content is fine, however, few more details would be helpful for the reader to better assess the limitations of the study (Taking shortcuts: Great for travel, but not for reproducible methods sections, bioRxiv 2022.08.08.503174; doi: <https://doi.org/10.1101/2022.08.08.503174>)

Reply: We have expanded the methods section, as suggested, in places where we previously had citations. There is a lot of infrastructure work that goes into the background of performing these analyses, this has been described in the cited papers and it would take dozens of pages to repeat in detail. However, we agree that a modest amount of more detail should help. Substantial text has been added in describing the composite citation indicator, clarifying Science-Metrix methods, delineating single year and career-long data, clarifying country allocation for each author, and other aspects of the data and analysis.

*	The authors choose to not report the results from the career-long impact; even though they are largely similar, it would be nice to either add this information as supplement (and refer to it in the man text) or even add this data in the main text in an infographic or else. Incorporating this additional data could enrich the comprehensiveness of the study and provide readers with a more comprehensive perspective on the topic.

Reply: In the revision, we have expanded Table 1 to show both the single last year and career-wide results that testifies that they are very similar. We have also added a new Figure (Figure 1) and expanded the previous Figure (now Figure 2) so that both of these Figures show the results according to both career-long impact and single recent year impact in separate panels.

*	Full professor versus associate or assistant professor, are those universal positions one can hold? E.g., in Germany they have the concept of habilitation and junior Prof, but the requirements are different from other countries. Thus, do you consider someone early in their career into one of these categories or rather categorize under "other" if they are in the process of habilitation -I can imagine that this information is not easily accessible via LinkedIn or else..

Reply: As we clarify in the revision “We coded different academic titles into these three ranks, based on perceived equivalence, e.g. for UK readers may be considered the equivalent of associate professors.” For Germany full professors are those with professor title and Habilitation, but we had no such cases in our random sample of 200 authors. We did search meticulously for any and all mentions of academic ranks, but in the Discussion we have added that “We should caution that information available online on academic ranks may not be always up-to-date or complete, but any missingness is probably not affected by gender.”

*	Did you consider all authorships irrespective of the position (first, last, middle)? The order of authors listed on a scientific publication can carry significant weight and influence an author's reputation and career prospects. Women, in some cases, may be more likely to be listed as secondary or supporting authors rather than first or senior authors, which can impact their visibility and recognition in the academic community. This is not clear in the manuscript and results might be different when considering the position.

Reply: We considered all authorship positions in counting number of scientists of different genders. However, the calculation of the composite citation indicator that is used to identified the top-cited scientists gives a lot of weight to single, first, and last authorships, as opposed to middle authorship. We have thus added in the Discussion: “We considered all authorship positions in counting number of scientists of different genders. However, the calculation of the composite citation indicator that is used to identify the top-cited scientists gives a lot of weight to single, first, and last authorships, as opposed to middle authorship. It is possible that in some cases women may be more likely to be listed as secondary or supporting authors rather than first or senior authors (or not be listed at all) and this can impact their visibility and recognition in the academic community. Thus multiple forces may converge towards diminishing the chances of women becoming top-cited.”

Visualization(s):

*	The manuscript would highly benefit from visualizations e.g., the overall number of male versus female authors and the proportion of highly cited researcher in each group. Some of the information that is currently presented in the tables 1- 4 could easily be visualized for a better understanding.

Reply: In principle, we prefer the tabulated data, because they allow showing the exact numbers, therefore we have kept tables 1-4 (expanding also Table 1, as above). However, we agree that some added visualization may have benefits. Thus we have created a new Figure that shows box plots for the proportion of women in the 4 age cohorts for all 174 subfields, showing median, IQR, range and any outliers, with two panels (career-long impact and single recent year impact) and we have expanded the previous Figure (now Figure 2) to include similarly two panels and to offer more granular information.

*	A Flow chart including the number of excluded data based on unclear gender assignments or else would be helpful, especially for the inclusion or exclusion of countries as only countries with > 5000 authors are considered in your descriptive analysis. Visualizing the random sample and the respective exclusion in the flow chart would be helpful.

Reply: We did not exclude any data from the analyses and we did not exclude any countries, so a Flow chart would not give much information to have added value (the number of unclear gender is shown in table 1 already). Maybe the misunderstanding is because of the legend of Figure 1 where we show the data points “for the 53 countries that had more than 5,000 authors in that cohort.” We explain that the reason is that “Countries with fewer authors have too few top-cited authors and the gender ratio for top-cited authors would be driven by very small numbers.” Almost all of these countries have less than 2% of their authors be top-cited, thus they have just a handful of top-cited authors and may even have 0 counts for one or both genders.

*	Figure 1: It would be of interest to visually include the different countries into the figure, e.g., by including a color-code for high-income vs non-high income; the 53 countries could also be provided as a list in the supplement (including whether they were considered high- or non-high-income.

Reply: The vast majority of these 53 countries are high-income countries. We have redrawn the Figure (it is now Figure 2) to show with different color the high-income and subsets of non-high-income countries among these countries.

Supplementary Information:

*	The authors do link to the Elsevier Data Repository; however, it would be helpful for readers to provide a list of content and refer to any supplementary information within the main text. I think the repository contains valuable information and should be better assessable (consider curation and FAIR data principles).

Reply: We have expanded on the description of the contents of the tables that are the supplementary repository.

*	In line with the previous comment: there are few parts where data is not shown, this could be done via the supplement and linking to it. As an example: "Frequency of very high or very low female representation in different subfields was similar to that found in high income countries." Why is that data not shown and why making the separation and focus on the USA (apart from the fact that the authors are based in the states)? I would rather recommend showing the overall data for high income and then include a subsection with a focus on the US, rather than doing it the other way around. If there are any restrictions though, please mention this in the limitation section.

Reply: What we had stated was: “Frequency of very high or very low female representation in different subfields was similar to that found in high income countries (not shown). Detailed data on all 174 subfields appear in the Supplement.” The “not shown” may have been confusing, because the detailed data are indeed shown in the Supplement, therefore we have now deleted the “not shown” parenthesis. USA represents the lion’s share of authors and we feel it deserves a separate section, which does indeed follow after presenting the results for all high-income countries (and all non-high-income countries).

Results/ Discussion:

*	How was the country-level analysis performed if an author changed their affiliation over time e.g., from non-high income to high income? In the method section the authors state that they rerun the analysis for scientists from country X or Y. Could you please clarify how the assignment was done, maybe I missed this.

Reply: In the Methods, we had already clarified that “For country we took the first country an author publishes from as the best estimate (the country of his/her oldest published paper).” We have added now the further elaboration: “Some authors may move to different countries during their career and then there is no perfect solution on which country should represent them. However, gender bias has formative influence starting from very early age, therefore assigning these authors to the country of their oldest publication is probably the most appropriate choice. Information on country of birth (which may also be different from the country of oldest publication) is not available in Scopus and extremely difficult to find for most authors.”

*	In the random sample used for the manual analysis the authors stated in the methods section that they would describe whether top-cited scientists in the youngest cohort (publication age) were artifacts of older researchers, this is a very important point and I feel it is lacking in the results and discussion section. In general, the question relates to the position of an author within a paper -if a highly cited female first author is accompanied by a male senior author. I realize that this might be beyond the scope of this publication, however, a critical discussion regarding the diversity in the author list of a given publication would be highly appreciated.

Reply: Probably there is a misunderstanding here. The artifact of a few older researchers being included in the cohort of younger researchers is not due to situations where young first authors are accompanied by older senior authors. We clarify now that “This artifact arises because in a few cases the publications of an author are split in 2 or more different author ID files in Scopus that may contain papers covering different year spans. E.g. if an author who has published from 2000 until now has her publications split into a Scopus ID file that covers the papers published from 2000 until 2020 and a different Scopus ID file that covers the papers published after 2020, that second Scopus ID file will appear as if it belongs to a young author.” Such splitting is uncommon (as we report on precision, recall and accuracy of Scopus) and our manual in-depth validation also shows that it affects only a small percentage of authors.

*	Considering low-income or middle-income countries and the current publishing system, how large is the proportion of (highly-cited) authors in general considering high APCs? I think a brief overview of additional barriers (even if they cannot be explored in detail here) should be included in the discussion/ limitation section.

Reply: As had stated in the original submission “In the overall database, there was almost double the number of authors from high income countries (5,899,402) than from non-high income countries (3,171,720)” and “There is a rapid advent of massive research productivity in research and scientific publications in some non-high income countries like China (18-20), often with financial incentives that have attracted criticism (18). Therefore, the share of authors from non-high income countries has increased sharply. Among the youngest authors, those with a decade or less of Scopus-indexed publication history, half of them come from non-high income countries. In these countries, gender imbalances in the ability to reach the top-cited group remain much stronger than in high income countries. This poses an extra challenge to achieving equity in these countries, where research is often performed under suboptimal circumstances.”

*	In that context, funding disparities in high versus non-high-income countries as well as geo-political and social structures, traditions and cultural differences might play a major role and should be discussed in more detail. In that line, the gender gap (in STEM) is smaller in countries where women are more likely to major in those fields (Aldén and Neuman, Culture and the gender gap in choice of major: An analysis using sibling comparisons, Journal of Economic Behavior & Organization, 2022.). The manuscript would highly benefit from a discussion that highlights common issues in gender inequality countries that do not belong to the global north (Rose and Hardi; "With Education You Can Face Every Struggle": Gendered Higher Education in Iraq and Iraqi Kurdistan - Part Three: The Gender Problem). Highlighting these potential (additional) causes for the existing imbalance can help to raise awareness and should not be detangled from the inequity in authorship and career prospects.

Reply: thank you for these excellent suggestions. We have accordingly cited these references and added: “Previous work has shown that the gender gap in science, technology, and medicine fields is smaller in countries where women are more likely to major in those fields (ref. Aldén and Neuman, Culture and the gender gap in choice of major: An analysis using sibling comparisons, Journal of Economic Behavior & Organization, 2022.). Moreover, there are local factors and barriers in each low- and middle-income country that shape the characteristics of its workforce and the extent of distortion from gender bias (ref. Rose and Hardi; "With Education You Can Face Every Struggle": Gendered Higher Education in Iraq and Iraqi Kurdistan - Part Three: The Gender Problem).”

Reviewer #2:

This study performs a large-scale quantitative analysis of citation rates for authors of different gender in across different scientific subfields and at different stages of their independent research career. The analysis was applied to a dataset previously generated by this group, using a previously developed citation metric. New to this study, the authors used an automated gender identification algorithm to label authors in their dataset and compare citation rates based on these labels. They report an overall increase in representation of women researchers over time and an increase in the relative representation of women in the group of top-2% cited researchers. However, the degree of representation by women in these groups varies substantially across fields.

This study addresses an important, challenging question with a new and valuable dataset. However, it is confusing that the authors use no statistical tests to substantiate their results. In a broad sense, the conclusion that women are generally underrepresented in research but the degree of their representation has been increasing over time is not surprising. If the authors had made claims that did not align with a confirmation bias, these results would be rejected without statistical tests supporting the claims. In particular, statements about differences between fields and changes over time should be qualified or backed up with statistical tests.

Reply: Thank you for the kind consideration of our work. We have added some statistical testing of differences, where it may strengthen the manuscript, following your suggestions.

MAJOR CONCERNS

1. L. 173-74. "We avoided formal statistical testing…" The logic of this statement is not clear. Statistical tests can be used to assess results in exploratory studies. If claims are to be made about numerical differences, it seems quite reasonable to back up those claims with a significance test. If the goal here is simply to publish the expanded dataset with gender labels, that may be acceptable on its own. In this case, however, claims about numerical differences between groups should be qualified as anecdotal and not statistically supported. One might argue that the results are so obvious that no statistics are needed. But if that's the case, why bother with a numerical analysis in the first place? Alternatively, it seems like all the tables (1-3, at least) could benefit from fairly straightforward statistical tests. More specific points about these tables are below.

Reply: We have replaced the sentence “We avoided formal statistical testing given the exploratory descriptive nature of the analyses.” with “We performed exploratory statistical testing using analysis of 2x4 tables adjusting for trend with exact test and comparison of proportions. P-values are two-tailed.” P-values have been added to the main results of tables 1 and 3 that afford such testing and to the exploratory comparison of the random sample for those with academic ranks.

2. L. 269-281. "close manual verification" It is unclear what to take away from this section. At face value, the small subset shows no difference between gender groups. There is the observation that only a small fraction of authors sampled have independent faculty jobs, but is this surprising, given that most authors would complete some years of graduate school and often a postdoc before obtaining a faculty position? Can the authors make a comparison that shows time-to-independence is longer than for earlier generations of researchers? Some additional analysis is required to substantiate the claims made here.

Reply: Information on the time-to-independence is very difficult to obtain from manual perusal of online information, e.g. while it is possible to ascertain whether a scientist currently has a specific academic rank, it is far less common to have detailed information on when he/she attained that rank unless a very detailed CV is available which is the exception. Therefore, comparison with older cohorts is not meaningfully feasible with this approach. However, we have added that “In the past, the time to get a doctoral degree was shorter and scientists could become faculty and even full professors very soon after obtaining their doctoral degree. Lengthy graduate studies and multiple postdoctoral experiences are currently far more common before reaching independence (references added).” New references added are: 1. Institute of Medicine. The Postdoctoral Experience Revisited. Washington, DC: The National Academies Press; 2014. 2. Andalib MA, Ghaffarzadegan N, Larson RC. The postdoc queue: A labour force in waiting. Systems research and behavioral science. 2018;35(6):675–86. 3. Denton M, Borrego M, Knight DB. U.S. postdoctoral careers in life sciences, physical sciences and engineering: Government, industry, and academia. PLoS One 2022 Feb 2;17(2):e0263185.

LESSER CONCERNS

L. 179 "top-cited" Please state how this group is defined. The information is provided in the Methods, but it takes a certain amount of digging. It appears that top-cited means the subset of each group with the highest composite citation index (c-index) for papers published in the last years? Or citations in the last year? And ranked both with self-citations and without?

Reply: We have expanded the Methods to clarify this better. “For each of the 4 sets, we generated the list of the 2% top-cited scientists in each of the 174 subfields with the ranking based on the composite index. We generated data both for career-long impact (all citations received at any time for all papers published at any time) and for the citation impact in the most recent calendar year, in this case 2021 (citations received in 2021 to papers published in any time). This was done twice: with and without including self-citations (as done in our previous work).”

L. 183. Table 1. It would be helpful if the authors could provide percentages and R scores for the different groups. It is true that they can be computed from the raw numbers, but a reader would appreciated it if were reported in the table. A figure would be even better, if the authors are able to do it.

Reply: We have added R scores in the Table, and also added data with different definitions of top-cited (single last year and career-long). As stated in the replies to reviewer 1, we have also added a new Figure that shows in more detail the distribution of proportion of women in different age cohorts.

L. 201. A similar request applies to Table 2.

Reply: R scores have also been added now to Table 2.

L. 232. Table 3 seems to contain the key results of the study. It's clear that gender ratios are on average approaching 50%. But is the process accelerating? Slowing down? Are there significant differences or not between wealthy and less wealthy countries? The data as presented aren't convincing.

Reply: As above, we have added the results of formal statistical testing in Table 3.

L. 252-267. Figure 1. This figure it hard to interpret. Please add a line of unity slope to show where values on the y axis are greater than the x axis. Consider also plotting on log-log scale, as the small values are difficult to discriminate.

Reply: We have added to what is now Figure 2 a line of unity, as suggested. We tried log-log scale, but it does not help, it may distort the appearance of the data. We also show differ color markers for high-income countries and different sets of non-high income countries. The figure also has two panels with different determination of top-cited authors (career-long impact and single recent year impact).

L. 257 "9.87 in Italy" Typo? 9.87 seems very high.

Reply: Thank you for noticing, it is a typo, corrected now to 0.87.

Reviewer #3:

Summary

This study evaluates how the share of female authors among the top-cited 2% in Scopus in each of 174 research subfields has changed over time. Gender of authors is determined using the NamSor database. The authors find substantial heterogeneity across disciplines and subfields in women researchers' shares and the extent to which gender disparities in the top-cited 2% has closed over four publication-age cohorts: pre-1992, 1992-2001, 2002-2011, and 2012 or later. Additional analyses consider within-field heterogeneity in these disparities across countries. Finally, the authors selected a random sample of 200 researchers in the post-2011 cohort for additional manual web research (e.g., LinkedIn, Google Scholar, university websites, etc.) to evaluate differences by gender in academic rank and employment sector.

This paper asks an interesting question and presents plausible descriptive results. However, there are several aspects that need further clarification before publication.

Reply: thank you for the kind appreciation of our work. We have addressed the raised issues, as delineated below.

Major points:

1. The description of the 174 fields as "scientific" (and the authors as "scientists") throughout the paper is confusing, as readers might reasonably presume the subjects of analysis are all in the natural sciences or at least in STEM fields. As Table 2 shows, this is not the case: subfields also include humanities, family and gender studies, and so on. I would suggest either limiting the subfields discussed to natural sciences (including health sciences) for better comparability, or changing the presentation and discussion of the paper to better reflect this.

Reply: this is a fair point, but Science-Metrix describes itself as a classification of 174 subfields of science (and even its name conveys the same). To avoid confusion, we have expanded as follows to clarify this potentially confusing point upfront: “…in each of 174 scientific subfields, as defined by the Science-Metrix classification (11). These subfields cover all types of science, technology, and (bio)medicine as well as scholarly disciplines on the study of humanities and social disciplines. We use summarily the terms “science”, “scientific fields”, and “scientists” in the paper to cover all these diverse types of scholarship, even though, strictly speaking, some of these authors may not see themselves as “scientists”.”

2. As Table 2 also shows, the total number of published researchers varies tremendously across fields. Weighting subfields like Folklore (86 researchers post-2011), Drama & Theater (181), Art History (257), and Gender Studies (503) equally with subfields Public Health (18,050), Psychiatry (18,626), and Education (23,905) can yield misleading conclusions about the extent to which the broader research enterprise is approaching gender balance. Moreover, I also downloaded the full classification subfield list for Science-Metrix, and while e.g. medical topics appear to be reasonably disaggregated (allergy, pediatrics, ob/gyn, endocrinology, etc.), others (e.g., economics) are not. Different citation norms, publication frequencies and peer-review timelines across true subfields of a discipline can lead to differences in citations. At a minimum, I would recommend combining similar small fields so that the number of unique researchers is more similar across "subfields".

Reply: The number of authors classified within each subfield is indeed extremely variable, but this is a reflection of reality of science and scholarship: some subfields have far more people publishing papers on them than others. Science-Metrix is an established, validated system and it would be imprudent to merge on our own initiative some subfields or try to split some subfields into smaller groups. Any merging or division would be arbitrary and non-validated. Different subfields have differences in citation rates, and this is accounted for by the subfield division. Merging subfields would make the identification of top-cited authors more biased. It is also possible that smaller subgroups within a single subfield may have different citation rates, but again any subdivision of a given subfield would be arbitrary and non-validated.

3. For fields with very detailed subfields, is it possible that a given highly cited researcher is showing up in multiple subfields' top 2% lists? I don't know about Scopus and Science-Metrix article classifications, but Scimago's similar system classifies many journals in more than one subfield. I also don't see, though perhaps I missed it, whether the subfield classification of authors is based on the journals in which they published or article-level subfield tags. If the former, I would wonder how high-impact scientific journals like Science and Nature are categorized.

Reply: We clarify that “Each scientist may have published papers in more than one subfields, but eventually he/is classified into a single dominant subfield, the one that has the highest percentage among his/her papers. The utilized Science-Metrix classification uses allocation each journal in a single subfield, except for multidisciplinary journals where the articles may be split into multiple subfields.”

4. My own small-N of NamSor suggests it may systematically misclassify contemporary female names as male, and performs very poorly with Vietnamese names. I am in a male-dominated field, and entered the names of each of my 32 students this fall as a test case. Of these, all male students were correctly classified, with probabilities 95% or higher. Among the 14 women, fully half would either be excluded from this study or misclassified as male. Four of the women are Vietnamese, and of those, 3 would be excluded due to "unclear" gender using the 85% cutoff. Half of the 8 native-US women (all with Anglo / Western European names) were misclassified as men, with probabilities 86%, 93%, 95%, and 99%. Now consider the following thought experiment: what if parents who give female children gender-neutral or historically-male-typed names raise children who are more likely to disregard or actively challenge gender norms about degree fields? This paper's method would then systematically undercount the share of women researchers in male-dominated fields.

Reply: While we don’t doubt the results of your small-N sample, it is unlikely to be representative of the publishing scientific workforce. Our randomly selected and thus representative samples of 100 authors assigned to male gender by NamSor and 100 authors assigned to female gender by Nam Sor showed only 3 mis-assignments (three people who were men had been considered to be women by NamSor). Therefore the overall error rate is likely to be very low. However, for names of particular ethnicities, the accuracy may be less. The proposed thought experiment is interesting, but highly speculative and we doubt that career choice is so strongly related to name choice. We have acknowledged nevertheless that “Moreover, even with a >85% certainty selection threshold, some people will be assigned to the wrong gender by NamSor. This wrong assignment happened nevertheless in only 6 of 200 randomly selected authors examined in depth. It is possible that the risk of mistaken gender assignment varies across fields and it may be more likely to reflect women being assigned to male gender than the opposite. If so, this may cause some underestimation of the percentage of women in some countries.”

5. It's not clear what the country analysis is meant to portray, if authors' country is set based on the first observed publication. For many natural sciences fields, this likely represents the location of the author's graduate program or postdoc, which (in some fields more than others) might not correspond to their national origin or where they subsequently work. At minimum, more care is needed in discussing the meaning of these results. It could also be interesting to evaluate whether authors who publish in different countries over their careers have higher citations (e.g. due to networking) vs those who remain in the same country.

Reply: This is an interesting point. As replied also to reviewer 1, we have added in the Methods: “Some authors may move to different countries during their career and then there is no perfect solution on which country should represent them. However, gender bias has formative influence starting from very early age, therefore assigning these authors to the country of their oldest publication is probably the most appropriate choice. Information on country of birth (which may also be different from the country of oldest publication) is not available in Scopus and extremely difficult to find for most authors.” Given the dynamics of brain drain (where talented scientists from disadvantaged countries move to countries with better resources), it is very likely that authors who have published from multiple countries have higher citations than those who stayed in the original country, but tracking the country itinerary through Scopus is not efficient or accurate, plus demonstrated this very likely association between mobility and citations would be not relevant to our analysis of gender differences.

Minor points:

*	The pre-1992 cohort is likely to have more citations for men because the window for possible citations can be arbitrarily long, and men dominated most fields before 1980. I suggest excluding this cohort, or using a similar decade cutoff as for other cohorts, for better comparability.

Reply: In the main analysis where we focus on citations received in the latest year, older scientists who started publishing before 1982 are actually at a disadvantage, because the citation rate to their old work most likely has declined. In the analysis that focuses on citations received career-long, maybe the bias would be in the opposite direction. Overall, the two analyses show little difference (comparative data are shown now in Table 1) thus we would not want to exclude or curtail the breadth of the year span covered from our analysis.

. *	On p. 4, "there is evidence that citations are misused and gamed". I'm sympathetic to this point, but the authors need to clarify what "gamed" means in this context, and if it's important, how/whether we can detect differences by gender in "gaming" citations.

Reply: Our analysis can only shown the large, science-wide picture of authorship and top-cited authorship, but we do not have the means based on these data to tell whether men or women game citations more. As we state in the introduction “Previous analysis has shown (6) that, overall, women tend to publish fewer papers than men and that the field-adjusted number of average citations received is modestly larger when first author is a man rather than a woman.”
