# Supplementary material for: Gender imbalances among top-cited scientists across scientific disciplines over time through the analysis of nearly 5.8 million authors
Source: PLoS Biol. 2023 Nov 21;21(11):e3002385. doi: 10.1371/journal.pbio.3002385 (PMC10662734; doi:10.1371/journal.pbio.3002385)
Supplement: Attachment — Submitted filename: responsesoct5.docx [file pbio.3002385.s002.docx]

a) Please change your title to make it more accessible and to include some idea of the impressive scale of your study. We suggest the following selection: "Analysis of nearly 5.8 million authors reveals differential correction of gender imbalance for top-cited scientists across scientific subfields over time" or "Analysis of nearly 5.8 million authors reveals large heterogeneity across scientific disciplines in the amelioration of gender imbalances among top-cited scientists over time" or (possibly the snappiest) "Gender imbalances among top-cited scientists across scientific disciplines over time through the analysis of nearly 5.8 million authors"

Reply: We have changed the title as suggested to “Gender imbalances among top-cited scientists across scientific disciplines over time through the analysis of nearly 5.8 million authors”.

b) The Academic Editor asked me to re-iterate his/her request from the previous round about RRIDs: "The only thing they did not address that was simple and practical was the addition of RRIDs, which I am assuming they simply did not understand because they did not read the instructions to authors. https://journals.plos.org/plosbiology/s/materials-software-and-code-sharing The authors used 3 major tools. These three are commercial tools therefore there are no good scholarly papers and there will certainly not be any public github repository that will point to the code. Therefore the addition of a persistent unique identifier, the RRID, is the only way to mark which tools were used when the tools are discontinued at some future point (statistically speaking 75% of tools would likely be around for two years after publication). NamSor Version or date of access (RRID:SCR_023935) Science-Metrix Version or date of access (RRID:SCR_024471) Scopus Version or date of access

(RRID:SCR_022559) Please ask the authors to include these RRIDs to the methods section with any additional version or date of access information."

Reply: We have added these RRIDs, as suggested.

c) We note that you have deposited the underlying in the Elsevier Data Repository, V1, doi: 10.17632/wwykk8d48g.1 - many thanks for doing so. However, I note that the associated licence is CC BY NC. I consulted our data team, and they tell me that the data underlying a data cannot have a more restrictive licence than our CC BY one. Please could you therefore (preferably) switch the Elsevier licence to CC BY; if this is not possible, please lodge a copy of the data in (e.g.) Zenodo and provide the Zenodo URL/DOI in the paper's Data Availability Statement and Figure legends (see next point).

Reply: We have changed the license to CC BY, as suggested, in a new version of the data posting that includes the key data (including those relevant for the Figures).

d) Please cite the location of the data clearly in both Figure legends, e.g. “The data underlying this Figure can be found in https://doi.org/10.17632/wwykk8d48g.1” or “The data underlying this Figure can be found in https://doi.org/10.5281/zenodo.XXXXX”

Reply: We have cited the location of the data as suggested.

e) Please make any custom code available, either as a supplementary file or as part of your data deposition.

Reply: there is no custom code.
